# Annotations

**Published:** 1896-02-29

**Authors:** 


### Annotations.

### A Cruel "Surgical Operation."

At Wakefield the other day Arthur Crossley and Robert Smith were charged with "nicking" two hackneys and a chestnut mare. The defendants pleaded that "nicking" was a "surgical operation." Now what is "nicking"? In the case under consideration it consisted, first, in making several cuts deep into the flesh on the under aspect of the tails of the animals; secondly, in attaching a rope to the extremity of the wounded tail in each case, passing the rope over a pulley fixed in the ceiling of the stable, and hanging a weight at the end of the rope; and, finally, in compelling the horses thus operated upon to stand on their feet, without lying down, for three weeks, so that a certain quantity of new tissue might grow in the "nicks" or wounds made in the tail, and thus compel the tail to stand permanently in a more or less horizontal, instead of its natural pendulous position. Let any feeling person imagine what all this means to a horse. The cutting is painful and cruel, the fixing of the wounded tail to the ceiling is still worse; but the order to make a horse thus wounded stand in his stable without lying down for three weeks is nothing less than diabolical. The Wakefield magistrates very properly fined Crossley and Smith.

### An Injudicious Definition.

The devising of definitions is no easy task. Politicians in their programmes, lawyers in their pleadings, even religions in their creeds find words a stumbling-block when they have to be strictly used, and their meaning accurately defined. We need not wonder, then, at the difficulty which has been experienced in understanding the meaning of the term "labouring" classes, as used in the Local Government Act, 1894; nor need we blame too heavily the somewhat lax definition framed by the Local Government Board as a guide to Parish Councils in the matter. Nevertheless, it is well to point out at once how unphysiological is the conclusion at which they have arrived, and how harshly it may act upon certain classes of the community. The law officers have decided that the expression "labouring population" means the population that, in substance, makes a living by manual labour; that it includes all, such as smiths, ploughmen, carpenters, artificers, workers in factories, and others, whose work is in the main manual, though knowledge and skill also are required; but that it does not include those whose work is in the main a matter of knowledge and skill, though manual labour also be required, such as nurses, cooks, postmasters, clerks, or tradesmen in general. The dividing line may be hazy, but the principle is as clear as it is unphysiological, viz., that he who has worked with his hands all day shall work with his hands still more, while he who has laboured with his brain till it is tired shall labour with it still or else be idle. The one saving gleam of common sense is that factory workers shall be considered labourers, but the futility of such definitions as would restrict the term "labour" to that particular class of labour which is principally manual is shown by comparing the work in many textile factories—a mere standing by a frame or a loom tying broken threads, or sitting in a comfortable room wrapping up parcels of lace or wool, with the labour of a clerk, who, in addition to the brain-exhausting effort of bookkeeping, is lifting heavy ledgers all the day. Yet the factory worker is to have his allotment while the clerk is not. Even in principle the distinction between brain toil and hand toil is bad, as applied to the question of allotments. If there is one aspect of modern industry worse than another, both for the body and the brain of man, it is the modern method of each man spending his life on one class of work. An all-round development of his powers is what a man should aim at; a more and more one-sided development is what civilisation offers him. The natural revolt against this is seen in the rush to cricket, to football, and to cycling among the highly specialised indoor workers of the day; but it is obvious that many a man who has toiled all day with his brains, and, from an instinctive craving for a physiological balance of effort between brain and muscle, toils again on his cycle in the evening, might just as well, and far more usefully, dig potatoes and plant cabbages. Yet the Local Government Board decides that those only are labourers who labour with their hands.

### Sunshine, Air, and Germs.

The results of a very interesting series of experiments made in the hygienic laboratory at Marburg, by Mr. F. F. Westbrook, a Cambridge University travelling scholar, have just been published in the *Journal of Pathology and Bacteriology*. Sunlight kills many forms of bacilli, it is stated and admitted. But how and under what conditions does the sun perform these beneficent functions? And does it sometimes fail to kill deadly germs; and if so, why does it fail? These are important questions, not from the point of view of a pure and comprehensive science only, but also from the practical standpoints of life-saving and disease prevention. On these questions Mr. Westbrook's experiments at Marburg throw a good deal of light. The experiments were made with cholera germs. Some of the germs were placed in test tubes containing cultivation broth. The tubes were all of the same size and shape, but contained different quantities of broth; and the bacilli when insulated were therefore in media of varying depths. Still further, one test tube was fully exposed to sunlight, whilst the others, though exposed to the same sunlight, were surrounded by rings of black paper of differing widths. The experiments were designed to bring out the facts whether sunlight alone, or sunlight plus the presence of abundance of atmospheric air, possesses germicidal properties. The results went to show that in considering the question of the sun as a germ killer we must take into account his heat as well as his light. Sunlight, with plenty of air surrounding germs, will kill them speedily and effectually. Sunlight, without air, does not kill them at all. Sunheat of average intensity, when air is excluded from germs, makes them multiply very much quicker than they would without it. The whole series of experiments afford beautiful confirmation of conclusions long empirically established, namely, that sunlight and abundance of fresh air are the most efficient preventives of all forms of bacillary or zymotic disease which science has yet discovered.

